# Micronucleus Test of Polycan™, β-Glucan Originated from *Aureobasidium*, in Bone Marrow Cells of Male ICR Mice

**DOI:** 10.5487/TR.2008.24.1.011

**Published:** 2008-03-01

**Authors:** Hyeung-Sik Lee, Hyung-Rae Cho, Kun-Ju Yang, Seung-Bae Moon, Bok-Ryeon Park, Hyun-Dong Shin, Hee-Jeong Jang, Lin-Su Kim, Sae-Kwang Ku

**Affiliations:** 12 Department of Herbal Biotechnology, Daegu Haany University, Gyeongsan, 712-715 Korea; 22 Marine Biotechnology Center 221, Glucan Corp. Research Institute, Busan, 617-763 Korea; 32 Department of Anatomy and Histology, College of Oriental Medicine, Daegu Haany University, 290, Yugok-dong, Gyeongsan-si, Gyeongsangbuk-do, 712-715 Korea

**Keywords:** Polycan™, β-Glucan, Micronucleus test, Genotoxicity, Mice

## Abstract

In this research the genotoxic effect of Polycan™ β-glucans originated from *Aureobasidium pullulans* SM-2001, was evaluated using the mouse micronucleus test. Polycan™ was administered once a day for 2 days by oral gavage to male ICR mice at doses of 1000, 500 and 250 mg/kg. Cyclophosphamide was used as a known genotoxic agent in a positive control group. The appearance of a micronucleus is used as an index for genotoxic potential. The results obtained indicated that Polycan™ shows no genotoxicity effect up to 1000 mg/kg dosing levels. In addition, it is also considered that there were no problems from cytotoxicity of Polycan™ tested in this study because the polychromatic erythrocyte ratio was detected as > 0.47 in all tested groups.

## INTRODUCTION

β-Glucan is a fiber-type complex sugar (polysaccharide) derived from the cell wall of baker's yeast, oat and barley fiber, and many medicinal mushrooms. The primary uses of β-glucan are to enhance the immune system (Czop, 1986), lower blood cholesterol levels (Lia *et al*., 1995) and to treat tumor (Gu *et al*., 2005). In addition, like other sources of soluble fiber, β-glucan is, according to preliminary studies, helpful in reducing the elevation in blood sugar levels that typically follow a meal. It produces this effect by delaying gastric emptying so that dietary sugar is absorbed more gradually, as well as by possibly increasing the tissue sensitivity to insulin. In turn, this will be helpful to control blood sugar levels in peoples suffer from diabetes (Braaten *et al*., 1994; Bourdon *et al*., 1999).

The preclinical safety tests for human trails of β-glucan have been studied in various types having different origins, and there are no toxicological evidences and no drug-drug interactions (Chihara, 1983; Feletti *et al*., 1992; Spicer *et al*., 1999; Delaney *et al*., 2003). Therefore, it has been used as ingredients of food, cosmetics and various types of drugs (Bais *et al*., 2005; Douwes, 2005; Plat and Mensink, 2005). However, the potential safety of β-glucan have still not been fully understood because they showed somewhat different characteristics according to origin (Seo *et al*., 2002).

Bone marrow cytogenetics, micronucleus test is a useful short-term technique for elucidating the mechanism as well as to identify the substances for their clastogenic and anticlastogenic activity (Renner, 1990). In Korea Food and Drug Administration (KFDA) guideline (2005-60, 2005), the genotoxicity should be tested prior to develop a new drug even though they have natural origin. Most of the mixtures having natural origin, genotoxicity has been performed using *in vivo* like micronucleus test (Kalantari *et al*., 2007).

Polycan$^{TM}$ is purified from *Aureobasidium pullulans* SM-2001 (half of the dry material is -1,3/1,6-glucans), a UV induced mutant of *Aureobasidium pullulans* and they showed somewhat different characteristics from other β-glucan derived from other origins (Seo *et al*., 2002). Recently the anti-osteoporotic (Shin *et al*., 2007) and anti-inflammatory (Kim *et al*., 2006, 2007) effects of

**Table 1.** Experimental design used in this study

| Group | Dosing materials (route) | No. of animals | Animal No. | Total dose (mg/kg) |
|---|---|---|---|---|
| G0* | Distilled water (oral) | 6 | G0-01~G0-06 | 0 |
| G1** | CPA (intraperitoneal) | 6 | G1-01~G1-06 | 70 |
| G2 | Polycan$^{TM}$ (oral) | 6 | G2-01~G2-06 | 1000 |
| G3 | Polycan$^{TM}$ (oral) | 6 | G3-01~G3-06 | 500 |
| G4 | Polycan$^{TM}$ (oral) | 6 | G4-01~G4-06 | 250 |

*Vehicle control; **Positive control; All test articles were taken once a day for coupled days; CPA, cyclophosphamide.

Polycan$^{TM}$ were at variance form the other purified β-glucans. Although mouse single oral dose toxicity test was performed (Lee *et al.*, 2005), there are no genotoxic studies about Polycan$^{TM}$ even if they have somewhat different characteristics.

The objective of the present study, therefore, was to obtain the genotoxic information about Polycan$^{TM}$, newly purified β-glucan from *Aureobasidium pullulans* SM-2001 and further clarify their safety for clinical use.

## MATERIALS AND METHODS

***Animals and husbandry.*** Thirty male ICR mice (6-wk old upon receipt, Jung Ang Lab. Animal Co., Seoul, Republic of Korea) were used after acclimatization for 9 days. Animals were allocated six per polycarbonate cage in a temperature (20~25°C) and humidity (30~35%) controlled room. Light : dark cycle was 12 h : 12 h and feed (Samyang, Seoul, Republic of Korea) and water were supplied free to access. Animals were marked by picric acid.

***Test articles and formulation.*** Polycan$^{TM}$ (Glucan Corp. Ltd., Pusan, Republic of Korea) is a brownish-sticky homogenous solution. It was stored in a refrigerator at 4°C. The test article was orally administered at a dosage volume of 20 ml/kg, once a day for 2 days by oral gavage to mice; total 1000, 500 and 250 mg/kg using distilled water as vehicle. Cyclophosphamide·$H_2O$ (CPA; Sigma, St. Louis, MO, USA) was used as an identified genotoxic agents in positive control group. CPA was dissolved in saline and once intraperitoneally administered at a volume of 10 ml/kg.

***Grouping and dosing.*** The animals were allocated into five groups 6 mice each. The fixed highest dosage level of 1000 mg/kg oral dosing was chosen in accordance to the results of single dose toxicity test in mice (Lee *et al.*, 2005), and 500 and 250 mg/kg was selected using the common ratio 2 according to the KFDA guidelines (2005). Control negative (taken vehicle) and control positive (CPA; 70 mg/kg-single treatment) were included by recommendation of KFDA guidelines (2005) and Organization for Economic Co-Operation and Development (OECD) guidelines (1997). The administered doses and schedule of these drugs are listed in Table 1.

***Observation of clinical signs.*** All abnormal clinical signs were recorded before and after dosing at least twice a day.

***Body weight changes.*** Body weights were measured once a day.

***Bone marrow preparation.*** All animals were sacrificed 24 h post administration using carbon dioxide, and bilateral femur was separated. Bone marrow preparations were made according to Schimid (1975). In brief, bone marrow cells were collected from aforementioned femur in 3 ml of inactivated fetal bovine serum (Gibco BRL, Grand Island, NY, USA), centrifuged, and smeared on slides. Preparations were dried, and fixed by submerging in absolute methanol (for 10~20 min). Fixed slides were stained as follows;

| | |
|---|---|
| May-Grunwald stain | 3 min |
| May-Grunwald stain (1 : 1 diluted) | 2 min |
| Giemsa stain (1 : 6 diluted) | 10 min |

***Observation and recoding of micronuclei.*** Slides were randomly coded and examined under × 1000 magnification by two different experts. Small round or oval shaped bodies, size of which ranging from 1/5 to 1/20 diameter of polychromatic erythrocytes (PCE), were counted as micronuclei (MN). Attention was given to discriminate micronuclei from artifacts. Results were expressed as the number of MNPCEs in 2000 PCEs. Mean number of MNPCE ± standard deviation was calculated for each treatment group. In addition, PCE/(PCE+normochromatic erythrocytes (NCE)) ratio were also calculated by counting 500 erythrocytes, for detecting the possibility of cytotoxicity (Heddle *et al.*, 1984).

***Statistical analyses.*** All MNPCE and PCE/(PCE + NCE) ratio were analyzed by Mann-Whitney U-Wilcoxon Rank Sum W test (MW test) compared to that of vehicle control. The result of statistical evaluation was regarded significantly when the P value was less than 0.05. In addition, the study was accepted when all of the PCE/(PCE + NCE) ratio are greater than 0.20 (Heddle *et al.*, 1984). Statistical analyses were carried out using SPSS for Windows (Release 6.1.3., SPSS Inc., USA).

## RESULTS

***Mortalities.*** No test article-treatment related unscheduled mortalities were detected in all tested doses during the observation periods.

***Clinical signs.*** During the observation period, no abnormal clinical signs were observed from Polycan™-treatment.

***Body weight changes.*** No meaningful changes on body weights were detected in CPA and all the tested doses of Polycan™ treated groups as compared to that of control negative group (taken vehicle only) (Table 2).

***Changes on MNPCE numbers and PCE ratio.*** Significantly ($p < 0.01$) increase of number of MNPCEs among 2000 PCEs was detected in CPA 70 mg/kg a positive control group. However, no significant changes on MNPCE numbers were detected in all three different Polycan™ treated groups tested as compared with vehicle control (Table 3). The PCE/(PCE + NCE) ratio in total 500 erythrocytes was detected above 0.47 in all tested groups including negative and positive control (Table 3).

**Table 2.** Changes on the body weights

| Group[1)] | | Day after dosing | | |
|---|---|---|---|---|
| | | Day 0[2)] | Day 1 | At termination |
| Male | G0 | 36.10 ± 1.20 | 36.25 ± 1.12 | 36.80 ± 0.51 |
| ICR | G1 | 35.50 ± 1.47 | 35.40 ± 1.63 | 35.83 ± 1.39 |
| Mice | G2 | 36.05 ± 0.93 | 36.60 ± 0.89 | 36.73 ± 0.79 |
| | G3 | 35.12 ± 1.41 | 35.73 ± 1.27 | 36.05 ± 1.83 |
| | G4 | 35.97 ± 1.27 | 36.28 ± 1.03 | 36.42 ± 1.54 |

Values are expressed as mean ± SD, g (n = 6).
[1)]Groups were listed in Table 1.
[2)]Start day of test article administration

**Table 3.** Changes on MNPCE numbers and PCE (PCE + NCE) ratio observed in mice

| Group[1)] | | MNPCEs/2000 PCEs | PCE(PCE+NCE) ratio PCE+NCE = 500 cells |
|---|---|---|---|
| Male | G0 | 1.17 ± 0.68 | 0.47 ± 0.01 |
| ICR | G1 | 40.00 ± 12.56* | 0.48 ± 0.02 |
| Mice | G2 | 1.17 ± 0.52 | 0.48 ± 0.01 |
| | G3 | 1.50 ± 0.45 | 0.49 ± 0.01 |
| | G4 | 0.67 ± 0.61 | 0.48 ± 0.01 |

Values are expressed as mean ± SD, g (n = 6).
[1)]Groups were listed in Table 1.
*$p < 0.01$ compared to that of G0 by MW test

## DISCUSSION

Micronucleus assays were first introduced in the early 1970's for the examination of genotoxic activity of chemical agents (Matter and Schmid, 1971; Heddle, 1973). The procedure is based on the observation that mitotic cells with chromatid breaks or incomplete exchanges or with malfunction of the spindle apparatus suffer from disturbances in anaphase distribution of their chromatin. After telophase, a sizable portion of this displaced chromatin is not included in the nuclei of the daughter cells but forms single or multiple micronuclei in the cell cytoplasm. The frequency of the appearance of micronuclei depends both upon the rate of chromosome breakage or loss and the rate of cell division (Von Ledebur and Schmid, 1973; Heddle *et al.*, 1984). Although micronuclei can occur in almost all dividing cells, mouse bone marrow is usually the tissue used for the micronucleus test, and any agent which induces chromosomal aberrations can also produce micronuclei (Heddle *et al.*, 1983, 1984).

Because of its simplicity and efficacy, the micronucleus test has become a popular and useful *in vivo* procedure for the detection of chemically-induced chromosome damage. The number of reports from micronucleus testing has increased dramatically in the scientific literature during the past decade (Ashby, 1985), and the value of this test for examining the mutagenicity and carcinogenicity of chemicals has been emphasized, particularly when it is used in combination with other cytogenetic assays (Heddle *et al.*, 1984).

In the present study, the genotoxic effects of Polycan™ were evaluated using the mouse micronucleus test. As the results obtained in the present study, Polycan™ shows no genotoxicity effect upto 1000 mg/kg dosing levels. The highest dosage used in the present study was selected as total 1000 mg/kg, based on the results of single dose toxicity test in mice (Lee *et al.*, 2005), and vehicle and positive control were added according to the recommendation of KFDA (2005) and OECD (1997) guidelines.

The PCE/(PCE + NCE) was used as index of cytotoxicity and the study was accepted when all of the PCE/(PCE + NCE) ratio are greater than 0.20 (Heddle *et al.*, 1984). The PCE/(PCE + NCE) ratio was detected as

> 0.47 in all tested groups including negative and positive control in the present study. That is no problem from cytotoxicity of the tested articles used in this work.

CPA is a widely used anti-neoplasic drug, employed either alone or in combination with other products (Grochow, 1996). The parent drug is biologically inactive, however after biotransformation by microsomal enzymes a number of active metabolites capable of alkylating nucleic acids (Miyauchi *et al.*, 1990), damage the chromosomes (through generation of free-radicals) and/or alkylating the DNA thereby producing mutagenicity (El-Bayoumy, 2001) were produced. In the present study, CPA used as a positive control, and it showed a significant increases of MNPCE ratios. This indicates that the experiment protocol and the results of the present study are acceptable, and no meaningful increases of MNPCE were reported up to 1000 mg/kg of Polycan™.

Based on the results, it is concluded that Polycan™ shows no genotoxicity effect up to 1000 mg/kg dosing levels. In addition, it is also considered that there were no problems from cytotoxicity of Polycan™ because the polychromatic erythrocyte ratio was estimated as > 0.47 in all tested groups.

## References

[CR1] Anonymous, Korea FoodDrug Administration (2005). Testing Guidelines for Safety Evaluation of Drugs (Notification No. 2005–60, issued by the Korea Food and Drug Administration on October 21, 2005).

[CR2] Ashby J (1985). Is there a continuing role for the intraperitoneal injection route of exposure in short-term rodent genotoxicity assays?. Mutat. Res..

[CR3] Bais D, Trevisan A, Lapasin R, Partal P, Gallegos C (2005). Rheological characterization of polysaccharide-surfactant matrices for cosmetic O/W emulsions. J. Colloid Interface Sci..

[CR4] Bourdon I, Yokoyama W, Davis P, Hudson C, Backus R, Richter D, Knuckles B, Schneeman BO (1999). Postprandial lipid, glucose, insulin, and cholecys-tokinin responses in men fed barley pasta enriched with beta-glucan. Am. J. Clin. Nutr..

[CR5] Braaten JT, Scott FW, Wood PJ, Riedel KD, Wolynetz MS, Brule D, Collins MW (1994). High beta-glucan oat bran and oat gum reduce postprandial blood glucose and insulin in subjects with and without type 2 diabetes. Diabet. Med.

[CR6] Chihara G (1983). Preclinical evaluation of lentinan in animal models. Adv. Exp. Med. Biol..

[CR7] Czop JK (1986). The role of beta-glucan receptors on blood and tissue leukocytes in phagocytosis and metabolic activation. Pathol. Immunopathol. Res..

[CR8] Delaney B, Carlson T, Zheng GH, Hess R, Knutson N, Frazer S, Ostergren K, van Zijverden M, Knippels L, Jonker D, Penninks A (2003). Repeated dose oral toxicological evaluation of concentrated barley beta-glucan in CD-1 mice including a recovery phase. Food Chem. Toxicol..

[CR9] Douwes J (2005). (1→3)-Beta-D-glucans and respiratory health: a review of the scientific evidence. Indoor Air.

[CR10] El-Bayoumy K (2001). The protective role of selenium on genetic damage and on cancer. Mutat. Res..

[CR11] Feletti F, De Bernardi di Valserra F, Contos S, Mattaboni P, Germogli R (1992). Chronic toxicity study on a new glucan extracted from *Candida albicans* in rats. Arzneimittelforschung.

[CR12] Grochow, L.B. (1996). Covalent-DNA binding drugs in The chemotherapy source book (Perry, M.C., Ed.), Williams & Wilkins, Baltimore, pp. 293–316.

[CR13] Gu YH, Takagi Y, Nakamura T, Hasegawa T, Suzuki I, Oshima M, Tawaraya H, Niwano Y (2005). Enhancement of radioprotection and anti-tumor immunity by yeast-derived beta-glucan in mice. J. Med. Food.

[CR14] Heddle JA (1973). A rapid in vivo test for chromosome damage. Mutat. Res..

[CR15] Heddle JA, Hite M, Kirkhart B, Mavournin K, MacGregor JT, Newell GW, Salamone MF (1983). The induction of micronuclei as a measure of genotoxicity: a report of the U.S. environmental protection agency gene-tox program. Mutat. Res..

[CR16] Heddle, J.A., Stuart, E. and Salamone, M.F. (1984). The bone marrow micronucleus test in Handbook of mutagenicity test procedures (Kilbey, B.J., Legator, M., Nichols, W. and Ramel, C. Eds.), Elsevier, Amsterdam, pp. 441–457.

[CR17] Kalantari H, Larki A, Latifi SM (2007). The genotoxicity study of garlic and pasipy herbal drops by peripheral blood micronucleus test. Acta Physiol. Hung..

[CR18] Kim HD, Cho HR, Moon SB, Shin HD, Yang KJ, Park BR, Jang HJ, Lim LS, Lee HS, Ku SK (2006). Effect of Exopolymers from *Aureobasidum pullulans* on formalin-induced chronic paw inflammation in mice. J. Microbiol. Biotechnol..

[CR19] Kim HD, Cho HR, Moon SB, Shin HD, Yang KJ, Park BR, Jang HJ, Lim LS, Lee HS, Ku SK (2007). Effects of β-glucan from *Aureobasidum pullulans* on acute inflammation in mice. Arch. Pharm. Res..

[CR20] Lee HS, Yang KJ, Shin HD, Park BR, Son CW, Jang HJ, Park DC, Jung YM, Ku SK (2005). Single oral dose toxicity studies of Polycan™, a β-glucan originated from *Aureobasidium* in mice. J. Toxicol. Pub. Health.

[CR21] Lia A, Hallmans G, Sandberg AS, Sundberg B, Aman P, Andersson H (1995). Oat beta-glucan increases bile acid excretion and a fiber-rich barley fraction increases cholesterol excretion in ileostomy subjects. Am. J. Clin. Nutr.

[CR22] Matter B, Schmid W (1971). Trenimon-induced chromosomal damage in bone-marrow cells of six mammalian species, evaluated by the micronucleus test. Mutat. Res..

[CR23] Miyauchi A, Hiramine C, Tanaka S, Hojo K (1990). Differential effects of a single dose of cyclophosphamide on T cell subsets of the thymus and spleen in mice: flow cytofluorometry analysis. Tohoku J. Exp. Med..

[CR24] Organization for Economic Co-OperationDevelopment Ed.. (1997). Guideline for the Testing of Chemicals TG No. 474. Mammalian Erythrocyte Micronucleus Test, July 21, 1997.

[CR25] Plat J, Mensink RP (2005). Food components and immune function. Curr. Opin. Lipidol..

[CR26] Renner HW (1990). *In vivo* effects of single or combined dietary antimutagens on mutagen-induced chromosomal aberrations. Mutat. Res..

[CR27] Schimid W (1975). The micronucleus test. Mutat. Res..

[CR28] Seo HP, Kim JM, Shin HD, Kim TK, Chang HJ, Park BR, Lee JW (2002). Production of -1,3/1,6-glucan by *Aureobasidium pullulans* SM-2001. Korean J. Bitechnol. Bioeng..

[CR29] Shin HD, Yang KJ, Park BR, Son CW, Jang HJ, Ku SK (2007). Antiosteoporotic effect of Polycan™, beta-glucan from *Aureobasidium*, in ovariectomized osteoporotic mice. Nutrition.

[CR30] Spicer EJ, Goldenthal EI, Ikeda T (1999). A toxico-logical assessment of curdlan. Food Chem. Toxicol..

[CR31] Von Ledebur M, Schmid W (1973). The micronucleus test — methodological aspects. Mutat. Res..

